# Supplementary material for: Genome-Wide Identification and Expression Analysis of the High-Mobility Group B (HMGB) Gene Family in Plant Response to Abiotic Stress in Tomato
Source: Int J Mol Sci. 2024 May 28;25(11):5850. doi: 10.3390/ijms25115850 (PMC11172549; doi:10.3390/ijms25115850)
Supplement: Supplementary file 1 [file ijms-25-05850-s001.zip › Supplementary Figures.pdf]

Supplementary figures

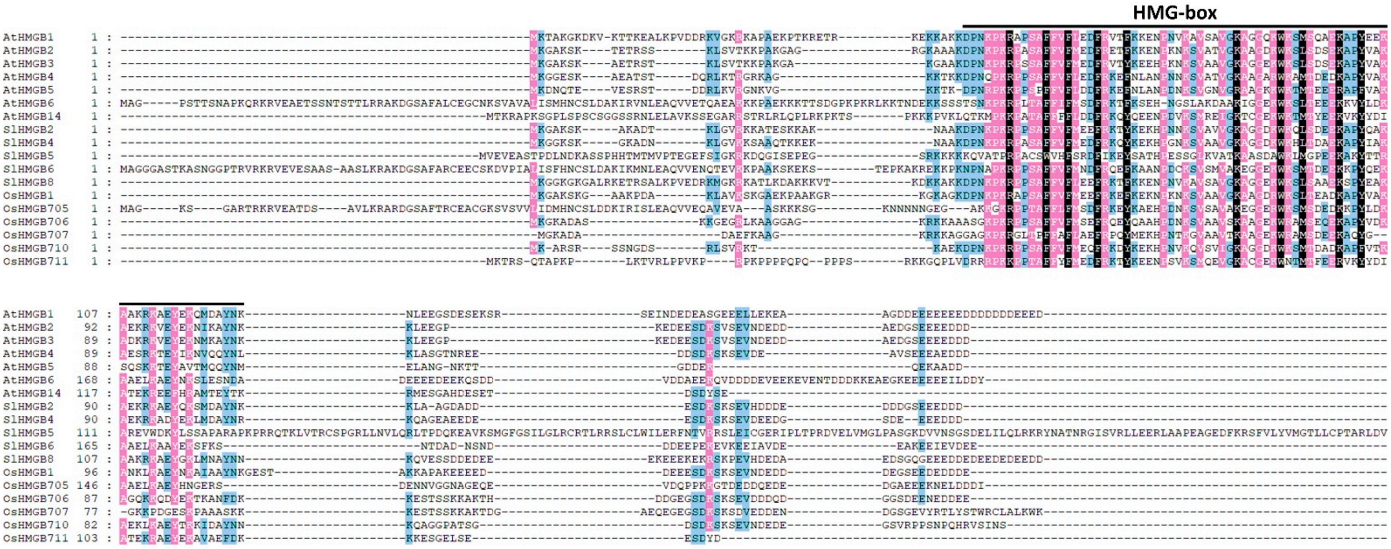

Figure S1. Multiple sequence alignment of HMGB proteins of group I in different species. The HMG-box domain is shown in the black lines. At, *Arabidopsis thaliana*; Os, *Oryza sativa*; Sl, *Solanum lycopersicum*.

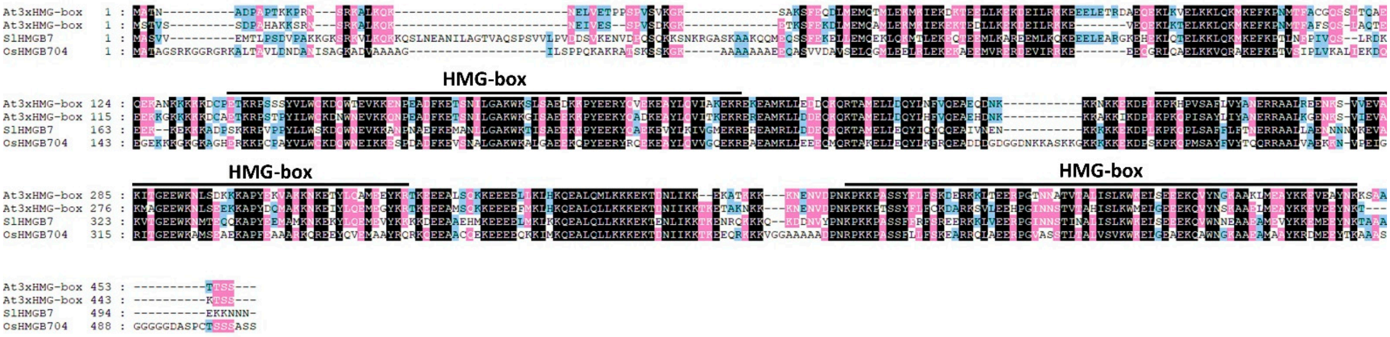

Figure S2. Sequence alignment of HMGB proteins of group II in different species. The HMG-box domains are shown in the black lines. At, *Arabidopsis thaliana*; Os, *Oryza sativa*; Sl, *Solanum lycopersicum*.

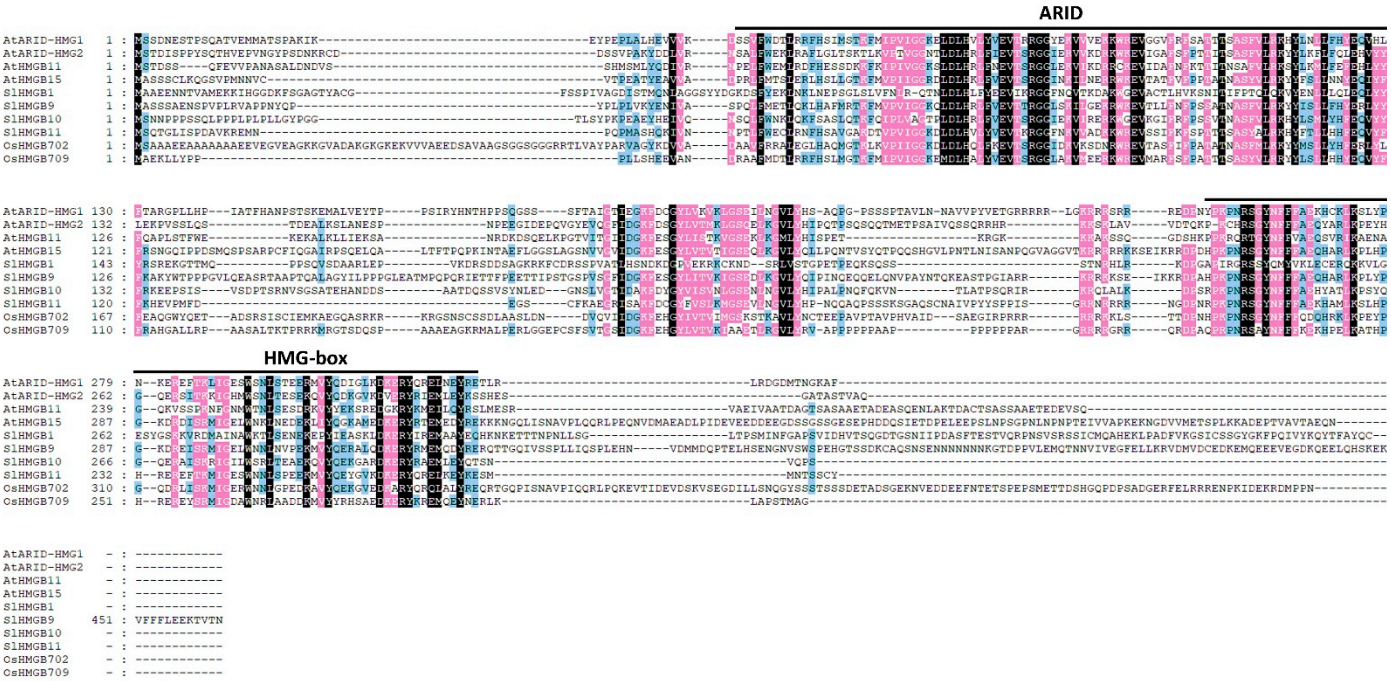

Figure S3. Sequence alignment of HMGB proteins of group III in different species. The ARID and HMG-box domains are shown in the black lines. At, *Arabidopsis thaliana*; Os, *Oryza sativa*; Sl, *Solanum lycopersicum*.

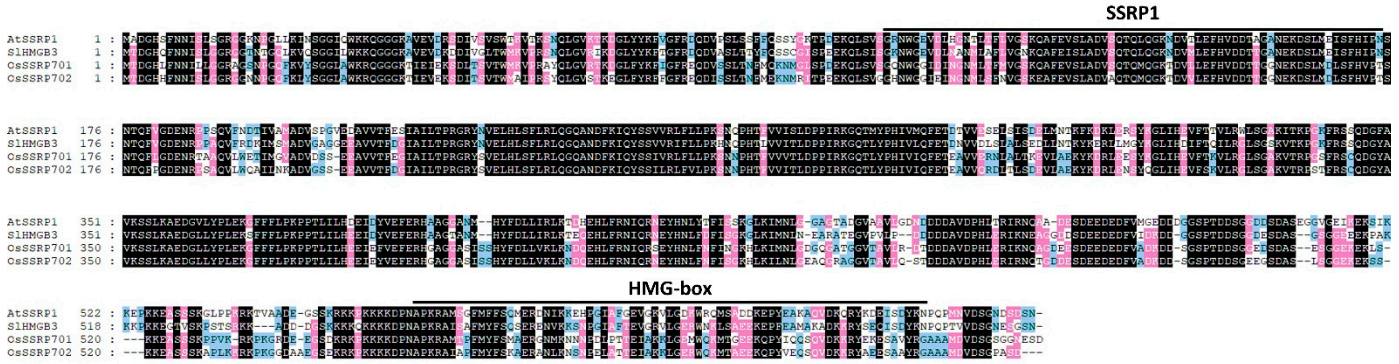

Figure S4. Sequence alignment of HMGB proteins of group IV in different species. The SSRP1 and HMG-box domains are shown in the black lines. At, *Arabidopsis thaliana*; Os, *Oryza sativa*; Sl, *Solanum lycopersicum*.

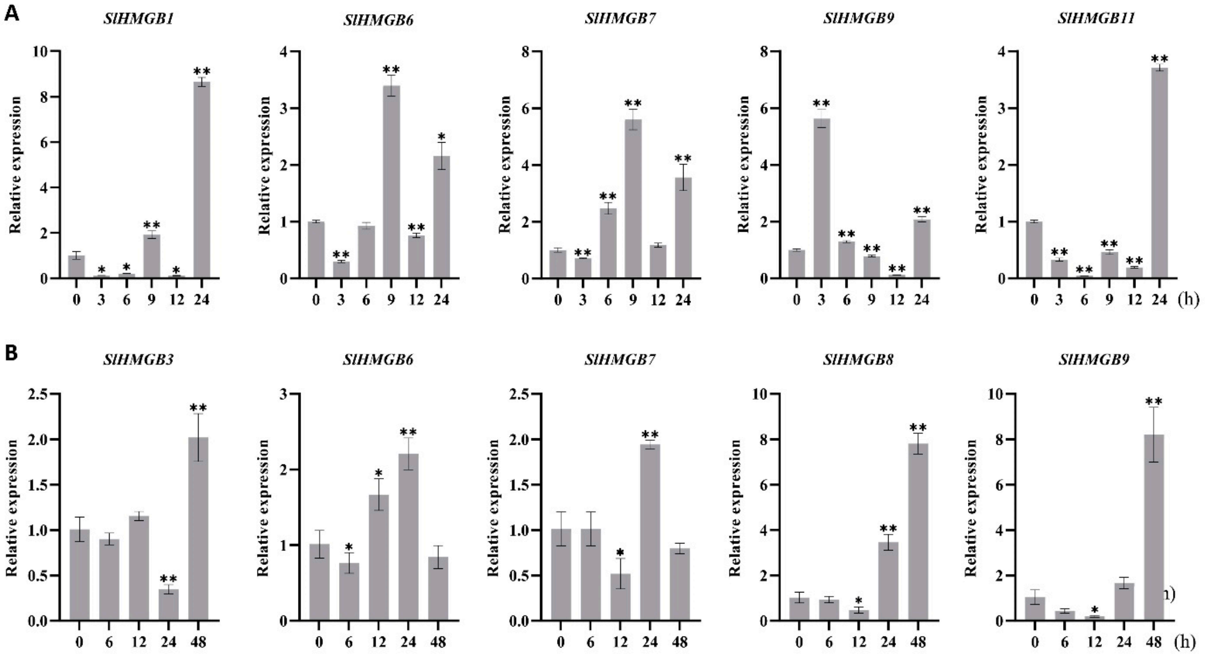

Figure S5. Disordered expression patterns of several *SlHMGB* genes under salt and drought stresses. (A) Relative expression levels of *SlHMGBs* under salt stress. (B) Expressions of *SlHMGBs* under drought stress. Each value is the mean ± SD of three biological replicates. Asterisks represent significant differences in gene expression between abiotic stress treatments (different time points) and Control (0 h) by Student's *t*-tests (* $P < 0.05$, ** $P < 0.01$).

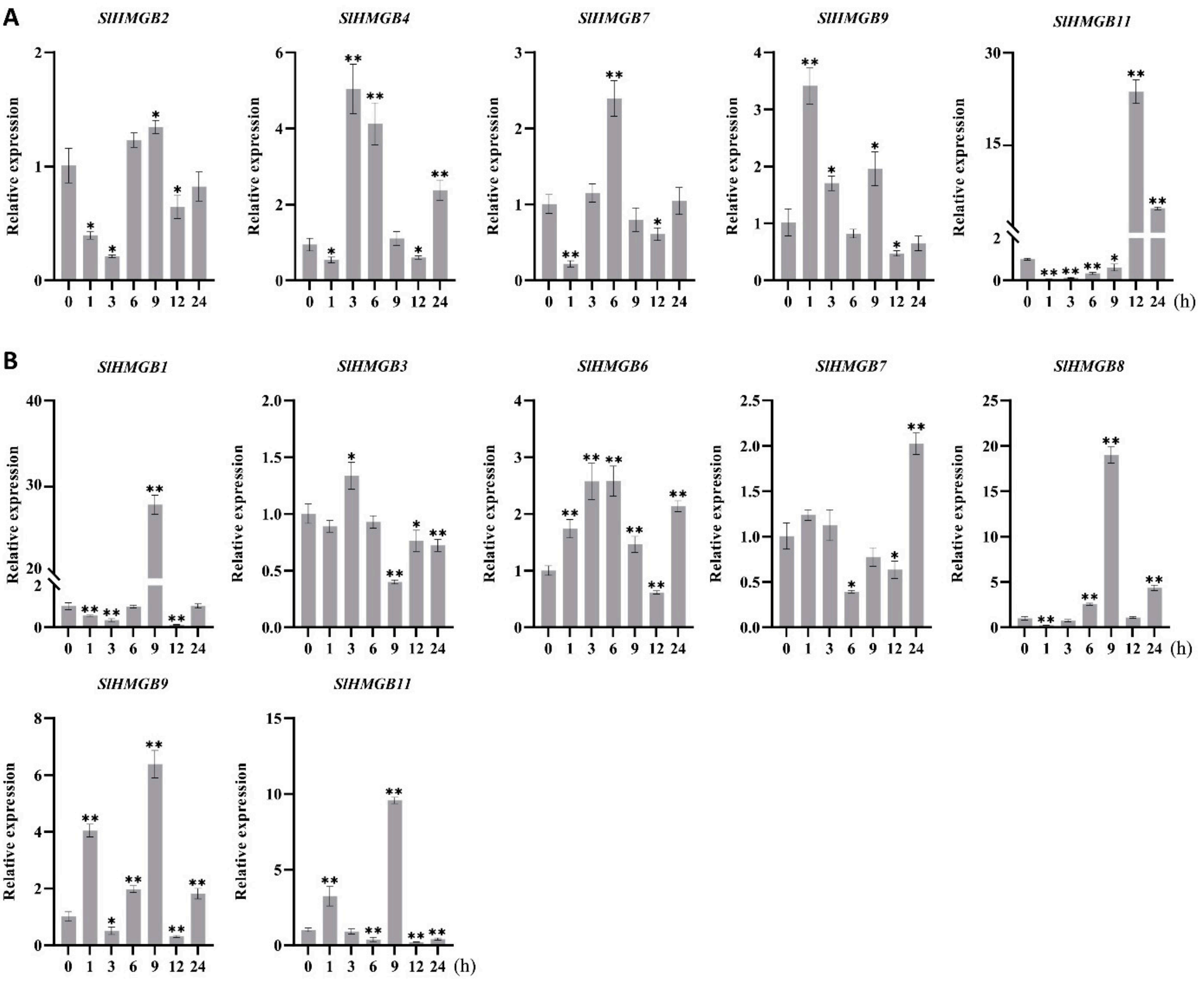

Figure S6. Disordered expression patterns of several *SlHMGB* genes under heat and cold stresses. (A) Expressions of *SlHMGBs* under heat stress. (B) Expressions of *SlHMGBs* under cold stress. Values are the means ± SD of three biological replicates. Asterisks represent significant differences between abiotic stress treatments (different time points) and Control (0 h) by Student's *t*-tests (* $P < 0.05$, ** $P < 0.01$).

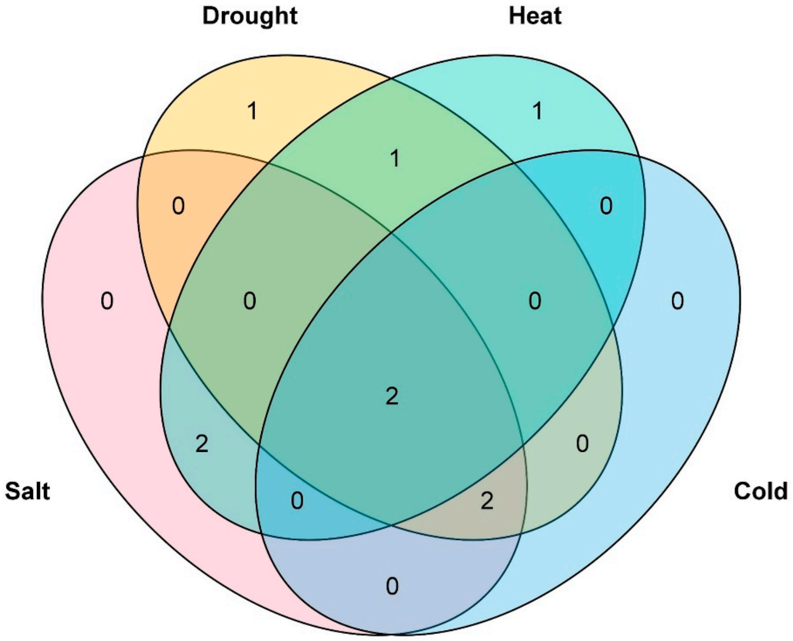

Figure S7. Venn diagram of *SlHMGB* genes involved in salt, drought, heat, and cold stresses.
